# Wound-Healing Studies in Cornea and Skin: Parallels, Differences and Opportunities

**DOI:** 10.3390/ijms18061257

**Published:** 2017-06-12

**Authors:** Anne Bukowiecki, Deniz Hos, Claus Cursiefen, Sabine A. Eming

**Affiliations:** 1 Department of Ophthalmology, University Hospital of Cologne, 50937 Cologne, Germany; anne.bukowiecki@uk-koeln.de (A.B.); deniz.hos@uk-koeln.de (D.H.); claus.cursiefen@uk-koeln.de (C.C.); 2 Department of Dermatology, University of Cologne, Kerpener Strasse 62, 50937 Cologne, Germany; 3 Center for Molecular Medicine Cologne (CMMC), University of Cologne, 50931 Cologne, Germany; 4 Excellence Cluster: Cellular Stress Responses in Aging-associated Diseases, CECAD, University of Cologne, 50931 Cologne, Germany

**Keywords:** cornea, skin, wound healing, inflammation, regeneration, repair

## Abstract

The cornea and the skin are both organs that provide the outer barrier of the body. Both tissues have developed intrinsic mechanisms that protect the organism from a wide range of external threats, but at the same time also enable rapid restoration of tissue integrity and organ-specific function. The easy accessibility makes the skin an attractive model system to study tissue damage and repair. Findings from skin research have contributed to unravelling novel fundamental principles in regenerative biology and the repair of other epithelial-mesenchymal tissues, such as the cornea. Following barrier disruption, the influx of inflammatory cells, myofibroblast differentiation, extracellular matrix synthesis and scar formation present parallel repair mechanisms in cornea and skin wound healing. Yet, capillary sprouting, while pivotal in proper skin wound healing, is a process that is rather associated with pathological repair of the cornea. Understanding the parallels and differences of the cellular and molecular networks that coordinate the wound healing response in skin and cornea are likely of mutual importance for both organs with regard to the development of regenerative therapies and understanding of the disease pathologies that affect epithelial-mesenchymal interactions. Here, we review the principal events in corneal wound healing and the mechanisms to restore corneal transparency and barrier function. We also refer to skin repair mechanisms and their potential implications for regenerative processes in the cornea.

## 1. Introduction

The cornea is the most anterior part of the eye and accounts for about 70% of its refractive power. The transparent nature and defined curvature of the cornea assure that light is focused and transmitted without scatter through the lens and onto the retina. Therefore, the tissue integrity of the cornea is of particular importance for clear vision. Eye injuries such as physical or chemical trauma or severe infections may result in permanent corneal damage leading to opacification and loss of visual acuity. Thus, rapid restoration of corneal integrity after injury is key to prevent intraocular inflammation that can cause permanent loss of vision or even loss of the eye itself.

Like in skin wound healing, inflammation is a fundamental process in corneal wound healing [1]. While a prolonged inflammatory response may exacerbate tissue damage, therapeutic suppression of the inflammatory response (e.g., by glucocorticosteroids) may also impair healing and might lead to a delay in epithelial wound closure [2,3] or promote infections [4,5]. This review will focus on the corneal immune system as an important factor in corneal wound healing and regeneration. We will first give an overview of corneal (microscopic) anatomy and physiology. Second, we will focus on corneal angiogenic and immune privileges, which are essential for corneal function and homeostasis. Finally, we will provide deeper insight into the cellular events in corneal inflammation, their consequences for non-immune cell function and the outcome of the healing response.

## 2. Corneal Anatomy

**Figure 1:**
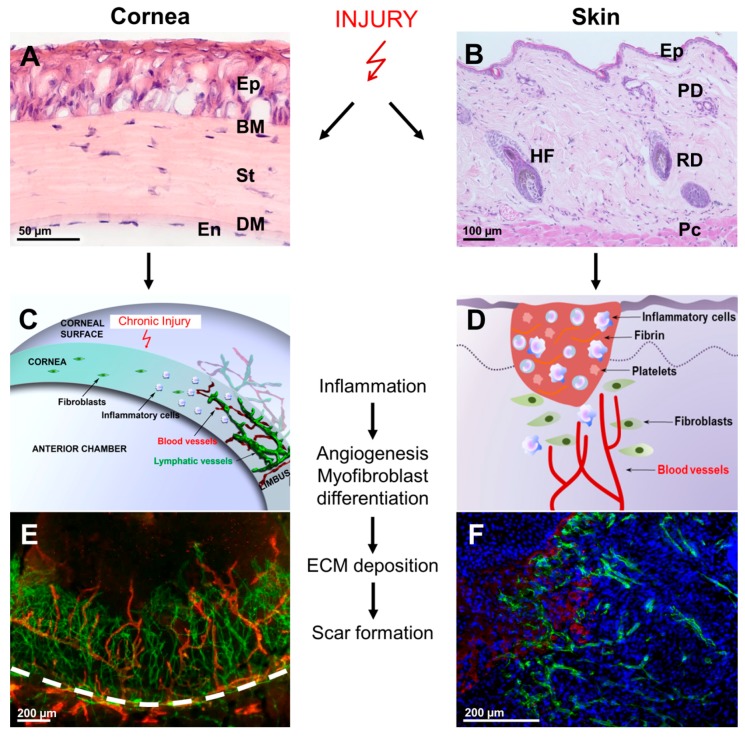
Parallels and differences in corneal and skin wound healing. Injury and wound healing in cornea and skin involves a similar sequence of events: inflammation, myofibroblast differentiation, extracellular matrix (ECM) deposition and eventually development of fibrosis. Yet, specific cellular responses, such as immediate keratocyte apoptosis after wounding, are unique events in corneal wound healing and presumably serve to avoid excessive corneal inflammatory reactions and opacification after tissue injury. Due to the physiological avascular nature of the cornea, hemostasis and activation of platelets are absent in corneal healing; however, both processes provide an important source of growth factors and cytokines in skin wound healing. Instead, in cornea, several of these factors (e.g., platelet-derived growth factor (PDGF)) are provided by epithelial cells [17]. Furthermore, to maintain its transparency, the cornea does not respond with the induction of capillary sprouts to minor injuries, which is essential to maintain good vision; however, chronic injury leads to an angiogenic response. (**A**) Histological view of the unwounded mouse cornea (HE staining); Ep: epithelium; BM: Bowman layer; St: stroma; DM: Descemet’s membrane; En: endothelium; (**B**) Histological view of the unwounded mouse skin (HE staining); EP: epidermis; PD: papillary dermis; RD: reticular dermis; Pc: panniculus carnosus; HF: hair follicle; (**C**) Infiltration of inflammatory cells into the physiologically avascular cornea and sprouting of blood and lymphatic vessels from the limbus after severe corneal injury (modified from Hos et al. [18]). Of note, corneal angiogenesis is a rare event and usually does not occur during wound healing. However, in severe and eye-threatening conditions, proangiogenic stimuli might overcome the corneal antiangiogenic mechanisms and lead to the secondary ingrowth of pathological vessels from the limbus into the corneal center. This is in contrast to skin wound healing, where capillary sprouts are important for an adequate healing response; (**D**) In skin wound healing, hemostasis, platelet activation and angiogenesis are key repair mechanisms. Similar to corneal wound healing, skin repair involves immune cell recruitment, activation and transdifferentiation of fibroblasts, which synthesize and remodel extracellular matrix and mediate wound contraction; (**E**) Murine corneal whole mount after experimentally-induced corneal injury and neovascularization (green: blood vessels, CD31 stain; red: lymphatic vessels, lymphatic vessel endothelial hyaluronan receptor 1 (LYVE-1) stain); the dashed white line indicates the limbal border. (**F**) Skin wound three days post-injury; immunohistochemical double staining for fibrin and blood vessels (blue: DAPI stain; red: fibrin stain; green: blood vessels, CD31 stain).

The cornea consists of five layers, with different regenerative capacities: (1) a stratified non-keratinizing squamous epithelial layer, which together with the tear film forms the outermost barrier of the eye; (2) the Bowman layer, an acellular collagenous layer beneath the epithelial basement membrane; (3) a collagen-rich stromal layer, which accounts for 80–90% of the corneal thickness in humans and 60–70% in mice; (4) Descemet’s membrane, which forms the basement membrane for the (5) endothelial cell layer on the posterior side of the cornea. The endothelial cell layer is a monolayer of polygonal cells that is in contact with the aqueous humor (Figure 1).

The corneal epithelium is developmentally derived from surface ectoderm and consists of stratified squamous epithelial cells. The epithelial surface is protected against damage and pathogens by the tear film, which also contains growth factors like epidermal growth factor (EGF) to promote epithelial regeneration [6]. Consisting of a lipid top layer to prevent evaporation, an aqueous middle layer and a mucin layer, the tear film protects the epithelial cells, washes away foreign particles and creates a smooth surface for clear vision. The tear film is uniformly spread across the cornea through the interaction of the mucin layer with the glycocalyx of the surface epithelial cells. Tight junctions connect the superficial cells to create an impenetrable barrier [7]. Beneath the superficial cell layer, the corneal epithelium consists of 2–3 layers of suprabasal wing-shaped cells, which rest on a single layer of basal cells attached to a basement membrane via hemidesmosomes. The basal cells are capable of proliferation and constantly replenish wing cells and superficial cells in the upper layers. In this process, proliferating basal cells move upwards and take on a more flattened shape. Superficial cells are continuously shed from the epithelial surface supported by eyelid blinking [7]. Similar to the skin, the epithelial basement membrane consists of various collagen types (IV, VII, XII, XV, XVII, XVIII), heparin sulfate proteoglycans, fibronectin, laminins and nidogens. Interestingly, the composition of the membrane has been shown to undergo changes during postnatal development and is not homogeneous, but considerably varies between the limbal region and the central regions above the Bowman layer [8,9,10]. The regions differ in collagen type IV and laminin isoforms [11], as well as fibrillin and tenascin expression [8]. The Bowman layer is located between the epithelial basement membrane and the anterior stroma. This layer is acellular and consists of randomly-arranged fibrils of collagen types I, III, V and VI [12,13]. It is well developed in humans, birds and higher mammals, such as cattle, whereas the Bowman layer is considerably thinner in rodents, such as mouse, rat, rabbit and guinea pig, while the collagen fiber diameters do not seem to vary among species [14].

While the epithelium provides a smooth surface to avoid light scatter, the stroma provides the corneas’ spheroid shape and accounts for its transparent nature and refractive power. The unique shape of the cornea generates only small spherical aberration, ensures that light is transmitted without scatter and is focused on the lens and retina. The stroma consists of tightly-packed collagen fibers (mainly consisting of fibrillary collagen types I and V), which form lamellae with parallel distributions. These collagen complexes are surrounded by small leucine-rich proteoglycans [15]. In the central cornea, the collagen lamellae are arranged in right angles relative to the fibers in neighboring layers, while towards the corneal periphery lamellae, they are orientated circumferentially [7]. This uniquely arranged network of collagen fibers is assumed to reduce light scatter and provide corneal clarity. The corneal stroma is devoid of blood and lymphatic vessels, but contains a dense network of autonomic and sensory nerve fibers. To ensure transparency, the central corneal axons lack myelin sheaths. As the primary cell type, the stroma contains sparsely-spread resting keratocytes, which continuously secrete collagen and proteoglycans to assure turnover of the extracellular matrix (ECM), although corneal collagen turnover is much slower than in skin [16]. Keratocytes are the major cell type involved in corneal repair. After injury, these mesenchymal-derived cells become activated and are able to transform into two phenotypes: fibroblasts and myofibroblasts.

The corneal endothelium like the stroma is derived from the neural crest and consists of a single layer of hexagonal-shaped cells resting on a basal membrane, named Descemet’s membrane. Descemet’s membrane is mainly composed of collagen type VIII, but also contains collagen type IV. The function of endothelial cells is of particular importance for corneal homeostasis, as endothelial cells feature a high number of Na^+^-K^+^-ATPases on their lateral membranes, creating an osmotic gradient that ensures that the cornea remains relatively dehydrated. The cornea ideally remains at a 78% hydration level, for which a sufficient number of endothelial cells is required to counteract corneal swelling and opacification. At birth, endothelial cell density amounts to 6000 cells/mm^2^ and decreases at a rate of about 0.6% a year. To preserve a sufficient dehydration rate, a minimum number of about 500–800 cells/mm^2^ is needed [7,19,20].

## 3. Distinct Regenerative Capacities of the Different Cellular Corneal Layers

The epithelium as the outer barrier is constantly self-renewing and has the highest regenerative capacity, as epithelial cells are replenished every 7–10 days. Epithelial stem cells reside in the limbal palisades and migrate towards the corneal center, where they differentiate to transient amplifying cells and basal cells [21,22]. Thus, renewal of epithelial cells not only involves a vertical movement of differentiating cells from deep to superficial layers, like in skin, but also a centripetal migration of stem cells from the limbus to the central cornea as they undergo differentiation [22]. The limbal stem cell niche is essential for corneal epithelial homeostasis, and patients suffering from limbal stem cell deficiency, e.g., due to severe chemical trauma to the limbal region or inherited diseases affecting limbal stem cells, often show severe and vision-limiting corneal alterations [23]. In these patients, overgrowth of the adjacent conjunctiva on the cornea (conjunctivalization) can frequently be observed, indicating that limbal stem cells are not only important to provide a cellular source for corneal epithelial turnover, but also to maintain the identity and barrier between various cellular compartments in the eye.

While stromal cell populations are quickly replenished, the challenge in stromal wound healing is to restore the exceptionally regular collagen organization, which is initially lost at the site of injury. This remodeling process and restoration of normal transparency can take several years [24]. Among all corneal layers, the endothelium has the lowest mitotic activity and lowest regenerative capacity. Therefore, to close small endothelial ruptures, the remaining endothelial cells will migrate and enlarge to remodel the monolayer. Consequently, cells will re-establish barrier function and resume their pumping activity [25]. As the endothelium is essential for the constant dehydration and thereby the transparency of the cornea, diseases or injuries leading to extensive loss of endothelial cells generally lead to corneal opacification due to excessive fluid accumulation and swelling. To date, the only treatment available to treat patients suffering from endothelial dysfunction or endothelial cell loss is corneal transplantation (keratoplasty) by procedures like DSAEK (Descemet’s stripping automated endothelial keratoplasty) or DMEK (Descemet’s membrane endothelial keratoplasty) where the endothelial cell layer and Descemet’s membrane (with or without stromal parts, respectively) are replaced by donor tissue.

## 4. The Corneal Angiogenic and Immune Privilege

In almost all organs, blood and lymphatic vessels form extensive networks that are vital for multiple functions, including the transport of nutrients, liquids, signaling molecules and cells. The cornea is one of the few tissues of the body that in its healthy state is entirely devoid of blood and lymphatic vessels. However, corneal homeostasis also depends on oxygen and nutrients, which the cornea receives via the tear film from the outside, the aqueous humor from the anterior chamber at the inner side and the limbal vasculature from the corneal periphery. Furthermore, in contrast to other tissues including the skin, the cornea usually does not respond with the induction of blood and/or lymphatic vessels to minor injuries and angiogenic stimuli. Such reactions would interfere with its transparency and result in vision loss. Therefore, the avascular state of the cornea, termed corneal angiogenic privilege, is essential for its function and is actively maintained.

In this regard, it was demonstrated that the corneal epithelium expresses soluble forms of the three major vascular endothelial growth factor (VEGF) receptors (sVEGFR-1, sVEGFR-2, sVEGFR-3), which are all assumed to act as decoy receptors to trap the key angiogenic growth factors VEGF-A, VEGF-C and VEGF-D, which might contribute to maintaining corneal avascularity [26,27,28]. Additional potent antiangiogenic molecules that are expressed in the cornea are angiostatin, endostatin, thrombospondin-1, thrombospondin-2 and pigment epithelium-derived factor [29,30,31,32]. These factors exert their antiangiogenic functions by blockade of vascular endothelial cell migration and/or proliferation or interfere with growth factor bioavailability. In addition to these molecules, the cornea also expresses inhibitory PAS domain protein (IPAS), a negative regulator of hypoxia inducible factor (HIF). It has been shown that IPAS inhibits HIF-mediated upregulation of VEGF in the cornea and supports to maintain corneal angiogenic privilege even under hypoxic conditions [33].

Although the corneal angiogenic privilege is essential for good vision and is therefore strictly and (in part) redundantly regulated, this condition is not invulnerable. In fact, severe inflammatory and potentially eye-threatening conditions can result in a massive upregulation of proangiogenic growth factors, which might overwhelm the antiangiogenic mechanisms of the cornea and result in a secondary ingrowth of both blood and lymphatic vessels from the limbal area into the corneal center (Figure 1). This angiogenic response immolates the transparency of the cornea and its functionality for the safety of the whole eye. Therefore, after the immune response is complete and barrier function has been achieved, corneal blood and lymphatic vessels need to resolve promptly to restore corneal transparency and functionality. However, in certain pathological conditions, corneal blood and lymphatic vessel regress very slowly, and may even persist and contribute to detrimental corneal diseases [34,35,36].

In addition to its angiogenic privilege, the cornea also belongs to the so-called immune privileged sites of the body [37]. In this regard, it has been shown that antigens (e.g., alloantigens) introduced into the anterior chamber result in the generation of antigen-specific regulatory T cells (Tregs), leading to tolerance and suppression of immune responses against these antigens. The immune privilege of the cornea has been attributed to several components: The healthy cornea contains only a few major histocompatibility complex (MHC) II-positive antigen-presenting cells (APCs), and corneal expression of MHC I molecules is reduced compared to other tissues. Furthermore, immunomodulatory factors, such as Fas ligand (CD95L) and programmed death ligand 1 (PD-L1), are present in the cornea at high levels and are able to suppress excessive immune responses by inhibiting T cell proliferation and inducing T cell apoptosis [38,39]. One additional and important factor responsible for corneal immune privilege is the so-called anterior chamber-associated immune deviation (ACAID). ACAID causes immune tolerance by antigen-specific downregulation of delayed-type hypersensitivity (DTH) reactions. This mechanism suppresses cell-mediated immune responses that are potentially caused by tissue damage. Antigens introduced into the anterior chamber are captured by APCs migrating to the marginal zone of the spleen, where they are involved in the activation of regulatory T cells, which subsequently suppress DTH immune reactions [40,41,42,43]. The spleen is indispensable for the development of ACAID, as it has been shown that removal of the spleen during introduction of various antigens into the anterior chamber prevents the development of ACAID and leads to rapid antigen-mediated rejection [44]. The immune and angiogenic privilege of the cornea are closely linked, as corneas with pathological corneal neovascularization often show a loss of their immune privilege in parallel [45,46]. In this context, it is generally accepted that the presence of pathological corneal blood and lymphatic vessels are the main risk factor for immunological graft rejection episodes after corneal transplantation [47,48].

## 5. Immune Cells in the Resting Cornea

Initially, it was assumed that antigen-presenting cells (APCs) were virtually absent from corneal tissue, which was believed to be one of the underlying mechanisms of the corneal immune privilege [49,50]. Since then, the view of the corneal cellular immune system has dramatically changed, and virtually all types of immune cells have been shown to be residing in the epithelium and stroma of the naive cornea. Immunohistological analyses in mice carried out with the pan-leukocyte marker CD45 could identify leukocytes located mostly in the corneal periphery, but also in the epicentral and central cornea [50,51]. CD45^+^ cells are distributed through the entire depth of the stroma, although most cells are either located in the anterior or posterior third of the stroma [51]. In bone marrow transplantation experiments after corneal irradiation, it was demonstrated that these myeloid-derived CD45^+^ cells are constantly reconstituted from the bone marrow, with 75% of cells being replenished within eight weeks [52]. However, one has to keep in mind that irradiation of the cornea might have substantially affected the turnover rate of these cells.

Macrophages stained positive for CD11b were shown to make up about 50% of the resident corneal leukocytes [51]. They are localized primarily in the posterior stroma and are uniformly distributed in the periphery and center of the cornea [50]. Recently, the origin of these corneal macrophages has been investigated in detail, considering their CCR2 expression profiles. The current concept divides macrophages and monocytes into a proinflammatory CX3CR1^low^ CC R2^positive^ type and a tissue resident, anti-inflammatory CX3CR1^high^ CCR2^negative^ type [53]. Liu and colleagues reported that CCR2^negative^ macrophages possibly originating from the yolk sac or fetal liver can be detected in the stroma starting from E12.5, while CCR2^positive^ macrophages in the cornea are presumably fetal liver- or bone marrow-derived and appear at later stages in embryonic development starting from E17.5. Additionally, this work could demonstrate that CCR2^negative^ macrophages in the cornea are, for the most part, replenished by resident macrophage proliferation, whereas a large proportion of the CCR2^positive^ population is continuously reconstituted from the bone marrow [54].

Langerhans-cells (LCs), an epithelial type of dendritic cell, are found in the corneal epithelium in the limbus region, periphery and central cornea. Notably, central cells have been described to be MHC II negative, while peripheral cells are mainly MHC II positive [55]. In rat cornea, slow-cycling MHC II^+^ cells have been identified in the limbal basal epithelium, which are putative LC precursor cells [56]. Similar to the LC distribution, stromal dendritic cells (DCs) are found in the periphery and center of the anterior stroma, with the central cells lacking MHC II expression [50,57]. These DC populations have been shown to be replenished by bone marrow-derived cells [58]. In this regard, it has also been shown that the fractalkine receptor CX3CR1 is involved in homeostatic homing of corneal MHC II^+^ cells to the cornea [59].

LysM-positive neutrophils are located around the limbal vessels in the periphery, but are not found in the central cornea [60]. Similarly, mast cells are found in the corneal limbus and conjunctival parenchyma, but not in the central cornea of adult mice. They are present in the developing cornea starting from E12.5 and accumulate around the corneal vasculature, during embryonic development. Interestingly, the distribution of mast cells is influenced by eye-opening, as cells in the central cornea have been observed to disappear after eye-opening and thus after the first contact of the cornea with external factors [61]. Although in earlier reports, CD3^+^ T cells have been reported to be absent from the cornea [51], by now, resident CD4^+^ and CD8^+^ T cells have been identified in the central and peripheral region of naive corneas [62].

## 6. Corneal Wound Healing

Corneal wound healing involves a complex cascade of cellular events of which we can only highlight some aspects in this article. Depending on the extent of the injury, cells of the epithelium, stroma and endothelium react differently to restore corneal integrity. Corneal repair is orchestrated by a complex network of growth factors that largely overlaps with those factors reported in skin repair (reviewed in: [63,64,65,66]).

### 6.1. Epithelial Healing

Epithelial healing in the cornea has been studied extensively. As the outermost layer, the epithelium is prone to injuries and needs to repair quickly to prevent infection of deeper layers. Small epithelial defects are usually covered within 24 hours (h). Epithelial injury and apoptosis of the injured cells leads to disruption of the attachment to the underlying basement membrane. Subsequently, cells from the wound margin rapidly respond with flattening and centripetal migration. As cell-to-cell adhesions are partially maintained, the defect is slowly covered by a sliding cellular sheet. This process is followed by proliferation and differentiation of basal cells to restore the cell layer. During epithelial wound healing, a temporary ECM facilitates epithelial migration to cover the wound. Fibrin, fibronectin and hyaluronic acid are some of the molecules identified in this matrix [67]. During the terminal phase of epithelial healing, the cells generate new hemidesmosomes for anchorage to the underlying layer [67,68]. Multiple growth factors, also identified as key mediators of epidermal repair in skin, are involved in epithelial healing, including EGF, transforming growth factor β (TGFβ), hepatocyte growth factor (HGF) and keratinocyte growth factor (KGF) [69].

Epithelial regeneration is also supported by corneal nerves, which regulate the blinking reflex and thereby the turnover of corneal epithelial cells. Furthermore, corneal nerves have been shown to provide epitheliotropic factors, such as substance P, calcitonin gene-related peptide (CGRP) and nerve growth factor (NGF), among others [70,71,72,73,74,75]. Patients suffering from degeneration of corneal nerves (called neurotrophic keratopathy), e.g., due to herpetic disease or systemic diseases such as diabetic polyneuropathy, often show delayed epithelial wound healing and might even suffer from persistent and potentially sight-threatening epithelial wounds that might lead to corneal perforation or serve as entry for, e.g., bacteria into the eye, with devastating consequences. Topical treatment with recombinant NGF was shown to promote epithelial healing [75,76] and is currently in clinical trials for the treatment of several diseases of the ocular surface, like dry eye disease and neurotrophic keratitis, and for the promotion of corneal nerve regeneration after refractive and cataract surgery. Besides NGF, several other growth factors have already entered clinical trials, including EGF, fibroblast growth factor (FGF) and insulin-like growth factor (IGF), with promising results [77,78,79].

### 6.2. Stromal Healing

The first response observed in the corneal stroma after epithelial damage is keratocyte apoptosis beneath the wound, which is assumed to be initiated by cytokines released from damaged epithelial cells, including IL-1α, IL-1β, Fas ligand and TNFα [80,81,82,83,84]. Keratocyte death results in an area beneath the wound site virtually devoid of cells. The remaining keratocytes become activated, differentiate into fibroblasts and begin to migrate to the wound site within 24 h after injury [67]. Using a rabbit model of transcorneal freeze injury, it was recently observed that during this migration process, the fibroblasts form connected streams aligned in parallel to the collagen lamellae, suggesting that lamellae provide “contact guidance” for fibroblast migration [85]. Subsequently, fibroblasts proliferate to repopulate the wound site. During stromal healing, their migration and activation are assumed to be mediated by several growth factors, including TGFβ, platelet-derived growth factor (PDGF), FGF-2 and EGF [85,86,87,88]. TGFβ and PDGF have also been identified as the main growth factors involved in the transdifferentiation from fibroblasts to myofibroblasts, which occurs in stromal wounds [88,89,90,91,92,93,94].

Myofibroblasts are identified by their expression of contractile α-smooth muscle actin (αSMA) stress fibers, which enable them to mediate wound closure and contraction [95,96]. Additionally, myofibroblasts are characterized by the expression of vimentin and desmin and reduced transparency, compared to quiescent keratocytes [97,98]. The number of myofibroblasts at the wound site seems to vary between different types of wounds: In incisional wounds, where wound contraction is fundamental for proper healing, myofibroblasts seem to be most abundant [99,100]. Studies indicate that myofibroblast are not solely generated by local fibroblast proliferation and transformation, but by a large proportion are derived from bone-marrow cells recruited to the cornea as observed to some extent in skin [101,102,103]. As soon as the myofibroblast/fibroblast-mediated stromal wound closure is complete, the number of myofibroblasts in the stroma slowly decreases. The following remodeling of the stroma to restore transparency can take several years. In this process, the disorganized repair matrix is slowly substituted by regular corneal ECM.

### 6.3. Endothelial Damage Response

Endothelial wound healing is in most situations limited to the reorganization and enlargement of the remaining endothelial cells, as in humans their mitotic activity is negligible. As soon as the cells close the damaged areas, usually after a few days, they resume their pumping activity and begin to secrete new basement membrane. After corneal injury, endothelial cells may also undergo a process of endothelial mesenchymal transition (EnMT), similar to epithelial-mesenchymal transition (EMT) observed in skin. During EnMT, the usually quiescent endothelial cells attain fibroblast-like characteristics and begin to proliferate. EnMT is characterized by loss of cell-junctions, loss of apical-basal polarity and actin-skeleton reorganization (reviewed in [104]). Apart from numerous other factors possibly involved, this process has been shown to rely on TGFβ, FGF-2, IL-1β and involves NFκB activation [105,106,107,108]. EnMT may lead to fibrous ECM deposition (so-called retrocorneal fibrous membrane) posterior to Descemet’s membrane. The consequences of EnMT are therefore considered undesirable, as it might result in loss of endothelial cells and corneal opacification due to ECM deposition [109]. Moreover, EnMT is also observed in ex vivo cultured endothelial cells, leading to a loss of endothelial cell characteristics during attempts of bioengineering endothelial grafts. Efforts to expand endothelial cells in vitro aim to inhibit the process of EnMT and preserve cell morphology for instance by using inhibitors of TGF-β signaling or the Rho-kinase/ROCK pathway [108,110,111,112].

## 7. The Fibrotic Response in Corneal Repair

Corneal injury extending into the corneal stroma causes fluid influx and fibrin deposition, leading to swelling and subsequent local loss of transparency. During corneal stromal repair, fibroblasts and myofibroblasts deposit multiple elements of the ECM including type III collagen, fibronectin, tenascin and glycosaminoglycans, facilitating the migration of fibroblasts [113]. Prolonged myofibroblast activation and ongoing deposition of repair matrix can cause long-lasting corneal opacification. In addition, the collagens and glycosaminoglycans produced in the early phase of the repair response show irregularities in their fibril size, arrangement and composition, which are likely to contribute to permanent opacification and scarring [114,115]. The contraction of repair tissue may also alter the curvature of the cornea and its optic properties. However, corneal stromal injuries do not always result in corneal scaring and permanent opacification. Smaller and superficial wounds often heal with restitutio ad integrum. The exact mechanisms leading to full restoration or scarring of the injured cornea are so far poorly understood.

It has been shown that epithelial abrasion alone does not activate the fibrotic response. Fibrosis is only observed in corneal injuries that include rupture of the epithelial basement membrane and wounding of the stroma [116]. Similar to dermal repair in the skin, PDGF and TGF-β are pivotal mediators of the corneal fibrotic response [117]. Due to the angiogenic privilege, platelets as critical source of PDGF and TGF-β are virtually absent, although their accumulation around the limbal vessels after wounding has been reported [118]. In the cornea, PDGF is produced by epithelial cells and (like TGFβ) has been detected in tears [119,120,121]. For controlling the impact of these factors on stromal keratocytes, the integrity of the epithelial basement membrane appears important, by creating a barrier for the growth factors produced by epithelial cells and derived from tears. In the case of basement membrane rupture, PDGF gains access to the stroma and subsequently acts on keratocytes, stimulating their migration and proliferation [122]. Likewise, it was shown that upon loss of the basement membrane, TGF-β2 produced by the epithelial cells can get in contact with the stroma and induces keratocyte activation [116]. Accordingly, Han and colleagues recently reported that epithelial cells produce exosomes that after rupture of the basement membrane gain access to the stroma. These exosomes subsequently fuse with stromal keratocytes and are able to promote their differentiation into myofibroblasts in wounds affecting epithelium and stroma [123].

Interestingly, similar to scarless healing in embryonic skin, also corneal wounds in the embryo heal without a scar. In the chick embryo, incisional wounds affecting epithelium, basement membrane and anterior stroma do not involve fibrotic response and scar formation. Intriguingly, the stromal response in the embryo does not involve enhanced keratocyte apoptosis or proliferation and re-epithelialization in these wounds is observed to be very slow compared to adult [124]. The underlying cellular mechanisms involved in this scarless type of healing and whether this alternative pathway can still be activated in adulthood remain to be elucidated.

An exaggerated fibrotic response after corneal injury may rarely result in hypertrophic scar or keloid formation, characterized by excessive fibrous tissue. Compared to skin, the development of keloids is a very uncommon event in the cornea following corneal trauma or disease. As opposed to hypertrophic scars, which are restricted to the initial site of injury, keloids may overgrow the initial lesion covering large parts of the corneal surface and are often observed to be recurrent. Keloids clinically appear as defined, white, elevated tissue and are characterized by a hyperplastic epithelium, disruption of the Bowman layer and contain disorganized stromal collagen fibers, as well as activated fibroblasts and may be paralleled by neovascularization [125,126,127,128]. Interestingly, also spontaneous keloid formations without previous corneal injury have been reported [129,130,131]. It is unresolved whether a predisposition for dermal keloid formation, as observed in patients from Asian or African American ethnicity, is associated with a higher risk to develop corneal keloid after corneal injury or surgery [131]. However, corneal keloids have been observed to occur in genetic syndromes such as Lowe’s syndrome [132,133] and Rubinstein–Taybi syndrome [131,134], the latter of which has been shown to be accompanied by dermal keloid formation, which may occur spontaneously or after preceding trauma in up to 24% of the patients affected [135,136,137].

## 8. Cellular Events in Corneal Inflammation

Knowledge acquired about wound healing and cellular infiltration into the cornea has mostly been provided by corneal injury models in rabbit and mice, including epithelial abrasion [138], intrastromal suture placement [139], chemical burn injuries [140] or corneal incisions (for a review, see [141]). As each of these injury models is characterized by a different wound healing response, kinetics and quality of inflammation, combining the results into an overall picture of cellular events after injury is a difficult task. The results obtained may vary among the nature of injury, species and strains [142]. In general, immune cell recruitment after corneal injury is mediated by proinflammatory cytokines released from epithelial cells and keratocytes at the injured site. Il-1, Il-6 and TNFα have been shown to be important mediators [143,144,145,146,147]. Being attracted by these and several other cytokines, recruited leukocytes from the limbal blood vessels enter the stroma and migrate towards the wound site [148].

Neutrophils are the first cells infiltrating the cornea after injury: they can be detected as soon as 2 h after injury and have been observed to enter the cornea in two major waves at 18 and 30 h after epithelial abrasion; as soon as 48 h after injury, their numbers normalize again [149]. Neutrophils seem to be involved in several processes in corneal wound healing, as in neutropenic mice, re-epithelialization after abrasion was observed to be delayed [149,150]. Moreover, the absence of neutrophils was reported to affect the recovery of the corneal nerves, which was attributed to their secretion of VEGF-A as a neurotrophic factor [151,152]. Interestingly, the recruitment of neutrophils into the cornea seems to be dependent on platelet accumulation and vice versa, as it was shown that depletion of platelets decreased neutrophil influx, and in turn, the depletion of neutrophils led to reduced numbers of platelets accumulating around the limbal vessels [117].

Comparable to myeloid cell recruitment in skin injury, shortly after neutrophils have entered the cornea, macrophages extravasate from the limbal vessels, infiltrate the stroma from superficial to deeper layers and migrate towards the corneal center [148]. Macrophages remove debris and apoptotic cells at the wound site, but have also been shown to be essential mediators of angiogenesis after severe and prolonged corneal injury. The newly-formed blood and lymphatic vessels presumably serve to supply oxygen, growth factors and immune cells and in turn mediate their clearance. During corneal inflammation, macrophages express high levels of VEGF-A, VEGF-C and VEGF-D, thereby inducing the proliferation of vascular endothelial cells [153,154]. In addition, studies indicate that macrophages are able to form lymphatic vessel-like structures de novo [155]. It has recently been reconfirmed that macrophages are capable of forming tubular structures, by demonstrating that they form non-endothelial vascular channels in a subcutaneous tumor model [156]. Macrophages also take part in corneal wound closure by secreting TGF-β to promote the differentiation of fibroblasts to myofibroblasts. The experimental depletion of macrophages in both cutaneous and corneal injury in mice leads to a delay in wound healing and a reduction of αSMA-positive fibroblasts [157], as well as a delay in epithelial closure after abrasion [53,158].

Corneal injury also leads to an increase in Langerhans cell numbers in the corneal epithelium. LC recruitment into the cornea upon central corneal cautery was shown to be mediated by IL-1, TNF and CCR5 signaling in mice [159]. In rats, centripetal migration of MHCII^+^ LCs from the limbus to the central corneal epithelium was observed 4 h after central cauterization. The majority of these cells were shown to be recruited from the limbal basal epithelium [160]. The rise in LC numbers at the corneal surface increases the antigen presenting capacity of the cornea, which is important to adequately respond to foreign antigens, which might have been introduced through the wound. This might also apply to stromal DCs: while most DCs in the corneal center are MHC II negative, in the case of inflammation, their MHC II and costimulatory molecule expression are induced [160,161]. Yet, little is known about the role of stromal DCs in corneal wound healing, although it has been shown that corneal DCs migrate together with the epithelial sheet to cover the wound after epithelial abrasion in mice. Consistently, the experimental depletion of DCs delays epithelial closure and alters epithelial gene expression [162,163].

Natural killer (NK) cells, which are rare in the naive, intact cornea, accumulate around the limbal vessels and infiltrate the corneal stroma from the periphery to the center. Their influx peaks at 24 h after epithelial abrasion [164]. Experimental depletion of NK cells in mice results in delayed epithelial wound closure and impaired regeneration of corneal nerves. Moreover, in the absence of NK cells, neutrophil numbers were observed to be elevated [164], while DC numbers were reduced [162], indicating that NK cells possibly play a role in orchestrating the corneal inflammatory response [164]. However, the mechanisms involved are not yet resolved. Similar to findings in skin wound healing, a possible role in modulating the corneal immune response is also proposed for γδ T cells. This type of T cell, recruited via ICAM1 and CD18, was shown to promote wound healing and influence neutrophil and platelet numbers in corneal inflammation [165,166,167].

To date, it is still an open question by which mechanisms immune cells are recruited to the injured cornea during the inflammatory phase. Several chemokines and their receptors have been identified in the inflamed cornea, yet their functional role in immune cell recruitment to the cornea is poorly characterized. For instance, the CXC chemokines CXCL1 and CXCL5 [149], as well as CXCL10, CCL7 and macrophage chemoattractant protein 1 (MCP-1) expression [164] were found to be elevated after epithelial abrasion in mice. Correspondingly, CXCL1, CXCL8 and MCP-1 mRNA levels were found to be elevated in human inflamed corneas [168]. CCL5 and MCP-1 have also been shown to be secreted by corneal keratocytes after stimulation with IL-1 or TNF [143]. Additionally, CCR7 and its ligand CCL21 were found to be upregulated in inflamed corneas, mediating MHC II^+^ cell recruitment [169].

For the recruitment of neutrophils, it has been postulated that recruitment is mostly mediated by P- and E-selectins, but also involves ICAM-1 and CD18 interaction [149], whereas myeloid cell/macrophage recruitment to the cornea is assumed to be (partially) mediated by the chemokine receptor CCR2 and its main ligand MCP-1 [170,171]. In the cornea, MCP-1 is expressed by epithelial cells, as well as keratocytes [171] and was shown to be upregulated after epithelial scrape injury [144]. Furthermore, MCP-1 introduced into the rabbit cornea in a corneal-micropocket-assay led to increased macrophage infiltration and angiogenesis [172]. Consistently, in CCR2- or MCP-1-deficient mice, macrophage recruitment was demonstrated to be impaired, while neutrophil numbers were not affected [170]. The latter is unexpected given the critical role of macrophages in neutrophil phagocytosis and clearance. The number of corneal CD11b^+^ cells was also reduced in a model of topical CCR2 antagonist treatment [173]. Correspondingly, in skin wound healing, it was shown that CCR2-mediated recruitment of VEGF-A-expressing macrophages is critical for the initiation of capillary sprouting during the wound healing response [174].

Although macrophage polarization has been extensively studied in cutaneous wound healing [1,174], macrophage polarization phenotypes have hardly been investigated during corneal repair. It has been shown that CCR2-positive, as well as CCR2-negative macrophages are required for proper corneal epithelial healing [53]. In addition, alterations in gene expression levels of inflammatory factors such as inducible nitric oxide synthase (iNOS), but also an impaired transition of macrophages to the M2-like phenotype are associated with impaired wound healing following epithelial injury [158]. An experimental study by Uchiyama and colleagues in a rat alkali burn model has shown that treatment with a peroxisome proliferator-activated receptor gamma agonist prevents excessive corneal inflammation and results in a decreased profibrotic response with less corneal opacification. Interestingly, this study also demonstrated that although the overall number of macrophages was decreased in the treatment group, M2-like macrophage numbers were significantly higher, suggesting a superior role of this macrophage subpopulation in corneal wound healing, after chemical trauma [175]. Although the literature suggests that alterations in macrophage numbers and polarization phenotypes are associated with impaired corneal wound healing, definitive evidence for a functional role is missing.

Apart from MCP-1, it was shown that stromal fibroblasts upregulate a variety of additional chemokines after stimulation with proinflammatory molecules such as IL-1α or TNFα. Among those are granulocyte colony-stimulating factor (G-CSF), monocyte chemotactic and activating factor (MCAF), neutrophil-activating peptide (NAP) and monocyte-derived neutrophil chemotactic factor (MDNCF) [144], which all might be involved in monocyte/macrophage and granulocyte recruitment and activation after corneal damage. Collectively, current findings propose that homing and inflammatory influx of leucocytes into the cornea are mediated by multiple recruitment mechanisms. As yet, the predominant mechanisms are not identified. Furthermore, it remains unclear how tissue resident immune cells are involved in the inflammatory response. Due to the corneal immune privilege, these mechanisms may possibly vary from those observed in other tissues. After the repair response is complete and corneal integrity is reestablished, inflammation also has to resolve rapidly. In this regard, it is still unclear how inflammatory cells sense that tissue integrity is finally restored and how these cells are removed from the cornea. The mechanisms involved in immune cell clearance from the cornea still remain to be investigated.

## 9. Stem Cells in Corneal Regeneration

The limbus region provides the primary niche for corneal stem cells [176]. This area contains both the corneal epithelial and mesenchymal (stromal) stem cells [176,177,178]; although the term limbal stem cells (LSCs) is exclusively used for the epithelial progenitor subpopulation. LSC biology has been extensively studied over the past decades, and already several years ago, LSC transplantation has been transferred to the clinic for the treatment of various corneal epithelial deficiencies (reviewed in [179,180]). Recent experimental studies showed that some cells (3–4%) in the adult corneal stroma subjacent to the epithelial basement membrane also express markers of ocular progenitor cells that are not present in differentiated keratocytes [178,181]. These cells, termed corneal stromal stem cells (CSSCs), have the ability to divide extensively, generate adult keratocytes and have the potential to give rise to several non-corneal cell types, such as cartilage or neural cells [181]. CSSCs express genes indicative for mesenchymal stem cells (such as BMI1, CXCR4, ABCG2), as well as genes indicative for pluripotent cells (such as SOX2, KLF4, OCT4, NANOG). Furthermore, CSSCs express genes that are present in early corneal development (such as PAX6 and Six2) and genes associated with neural development (CDH2, NESTIN, NGFR) [182].

Several studies have analyzed the function of CSSCs in the corneal steady state and after injury. In vitro, CSSCs can produce connective tissue including collagen fibrils when cultured under low-mitogenic conditions [183]. Furthermore, when cultured on parallel aligned nanofibers, silk or polycarbonate substrates, CSSCs produce layers of highly parallel collagen fibrils with uniform diameter and regular interfibrillar spacing similar to that of the normal corneal stroma [184,185,186]. An important function of CSSCs is their support of the LSC population in the limbal stem cell niche [187,188]. Furthermore, experimental studies in mice have shown that CSSCs have the ability to support stromal regeneration and prevent scarring [189,190]: injection of CSSCs into the corneal stoma of Lumican-deficient mice, which have opaque corneas due to increased corneal thickness and altered collagen organization, results in restoration of the normal corneal architecture and reestablishment of corneal transparency [190]. Moreover, the application of CSSCs to surgical wounds comprising the epithelium and the anterior stroma (which usually leads to the deposition of disorganized and opaque scar tissue) results in deposition of extracellular matrix with normal collagen organization and clear corneas [189]. In addition, CSSCs have been reported to modulate immune responses in the cornea [190].

Collectively, CSSCs have been shown to facilitate corneal stromal regeneration without scarring and excessive inflammation. Although the therapeutic use of CSSCs is still far behind the use of LSCs, novel CSSCs-based therapeutic strategies might open up novel and effective treatment strategies for corneal stromal wound healing in the future.

## 10. Concluding Remarks and Future Directions

Corneal transparency is essential for proper vision, and a detailed understanding of the mechanisms involved in corneal wound healing and regeneration is of great importance to treat patients suffering from corneal diseases. At present, epithelial regeneration can be supported relatively satisfactorily, and it is possible to treat superficial corneal scars, e.g., with laser therapy. However, for deep corneal scars or endothelial diseases, which are frequent, corneal transplantation is still the only option to restore clear vision. Nonetheless, due to the scarcity of corneal donor tissue or limited access to corneal surgery, corneal opacification is still one of the major causes of blindness worldwide [191,192,193].

Ongoing research aims to support corneal regeneration by stem cell-based therapies. To support epithelial regeneration, research focuses on the ex vivo expansion and subsequent transplantation of LSCs derived from limbal biopsies, as already performed to treat patients suffering from limbal stem cell deficiency. As mentioned earlier, studies using LSCs and CSSCs have demonstrated beneficial effects in several disease models, promoting epithelial and stromal repair [189,190,194]. Due to the limited availability of corneal stem cells and the potential risks related to biopsy excision from donor eyes, several non-ocular tissues have been investigated as potential autologous sources of epithelial-like cells [195]. Apart from potentially simplifying the process of cell harvesting and culture conditions, these approaches would avoid immune reaction to donor tissue in patients with bilateral corneal disease. Here, oral mucosa epithelial cells [196,197] and conjunctival epithelial cells [198,199,200], already in clinical use, have been shown to successfully support epithelial regeneration in limbal stem cell deficiency, though data on long-term outcomes are limited. Interestingly, alternative approaches demonstrate that corneal epithelial-like cells may also be transdifferentiated from dermal sources: skin-derived precursor cells, epidermal adult stem cells and hair follicle stem cells were shown to attain corneal epithelial-cell like properties [201,202] and improve corneal healing [203]. For stromal regeneration, it has been demonstrated that adult stem cells isolated from dental pulp may be differentiated to attain a keratocyte-like phenotype, secreting collagen fibrils and corneal proteoglycans [204].

Innovative approaches show that corneal epithelial cell-like or keratocyte-like cells can also be differentiated from induced pluripotent stem cells (iPSCs) generated from corneal and non-ocular cells that might be used in future treatment options [205,206,207,208,209]. In an experimental therapeutic approach, iPSCs generated from human corneal keratocytes enhanced corneal healing in rats, when topically applied to cornea after epithelial abrasion [210]. Moreover, a broad area of research focusses on mesenchymal stem cells (MSCs) derived from non-ocular tissues to promote corneal and skin regeneration. MSCs derived from bone marrow, adipose tissue, umbilical cord or dental pulp [211,212] were demonstrated to acquire both epithelial cell-like [213,214] and keratocyte-like characteristics [215,216,217] (see [195] for a systematic review). The therapeutic use of bone-marrow or adipose tissue-derived MSCs, by local injection or topical application in animal models of chemical burns, was shown to improve corneal healing and reduce corneal inflammation and neovascularization [218,219,220,221].

Ongoing efforts in the field of endothelial regeneration focus on the establishment of methods for the isolation, cultivation, expansion and transplantation of corneal endothelial cells, with promising results [222,223,224]. These techniques are novel approaches to substitute for corneal transplantation procedures like DMEK in patients suffering from endothelial cell loss or dysfunction. Conclusively, cell-based therapies have the potential to accelerate corneal wound healing, compensate for epithelial cell, keratocyte and endothelial cell loss in certain injuries and might promote the substitution of opaque repair tissue with regular transparent corneal ECM. Further research will reveal whether these novel approaches will be successful in supporting corneal regeneration.
